# Anti-inflammatory and anti-oxidative effect of pasterurised yoghurt in indomethacin induced gastric ulceration in male Wistar rats

**DOI:** 10.1016/j.heliyon.2024.e40770

**Published:** 2024-11-29

**Authors:** O.V. Ajayi, B.O. Omolaso, T.P. Omayone, A.G. Adeniran, A.O. Adu, O.E. Olorunfemi

**Affiliations:** a Department of Physiology, University of Medical Sciences, Ondo City, Ondo State, Nigeria; b Department of Physiology, Federal University of Technology, Akure, Ondo State, Nigeria

**Keywords:** Gastric ulcer, Inflammation, Anti-oxidants, Indomethacin, Omeprazole, Probiotics, Pasteurized yoghurt

## Abstract

**Background:**

Yoghurt, a fermented dairy product consumed by diverse cultures for centuries, has garnered significant attention from the scientific community due to its potential health benefits and remarkable versatility. This study investigated the anti-inflammatory and anti-oxidative effects of pre-treatment with pasteurized yoghurt in indomethacin induced gastric ulceration.

**Method:**

Thirty male Wistar rats were randomly assigned into five groups. Groups 1 and 2, received distilled water (1 mL/kg), groups 3 and 4 received pasteurized yoghurt at 50 % and 100 % concentration respectively, group 5 received Omeprazole (20 mg/kg). All treatments were given orally for 14 days. Groups 2–5 were thereafter, administered with Indomethacin (40 mg/kg) and rats were sacrificed 4 h later. The stomach was carefully dissected out, scored for ulceration and used for histological evaluation and biochemical assays (GSH, SOD, MDA and CAT). Blood was collected and used for Interleukin 10 (IL10), Interleukin 1-Beta (Il-1β), Tumor Necrosis Factor Alpha (TNF-α) and Total Anti-oxidative Capacity (TAC) assays.

**Results:**

Indomethacin administration resulted in severe ulceration of stomach as well as significant increase in MDA, IL-10, TNF-α and Il-1β. This was accompanied by a significant decrease in GSH, SOD and TAC when compared with control group. Pre-treatment with pasteurized yoghurt caused a significant decrease in the inflammatory parameters and reverse oxidative stress induced by indomethacin administration. Gross and histological observation of the stomach indicate that pre-treated with pasteurized yoghurt offers cytoprotection when compared to indomethacin alone group by reducing inflammation and infiltration of the inflammatory aggregates at the mucosa layer. Therefore, we recommend that the dietary supplementation of pasteurized yoghurt should be encouraged during the treatment of gastric ulcer.

## 1 Introduction

Gastric ulcer is a lesion in the mucosa of the stomach lining that penetrates through the submucosa and sometimes to the muscularis propria and extends more than 5 mm in diameter. When alterations occur to the defense mechanisms of the stomach, it can cause changes in the gastric mucosa which will eventually result in erosion and then ulceration [1]. Gastric ulceration is a benign lesion on the mucosal epithelium upon exposure of the stomach to excess acid and aggressive pepsin activity. It is the most prevalent gastrointestinal disorder ever known, accounting for an estimated 15 mortality out of every 15,000 complications yearly in the world [2].

The use of Nonsteriodal Anti-inflammatory Drugs (NSAIDs) is the second most common cause of Peptic Ulcer Disease (PUD) with *H. pylori* infection being the first. The secretion of prostaglandin normally protects the gastric mucosa. NSAIDs block prostaglandin synthesis by inhibiting the COX-1 enzyme, resulting in decreased gastric mucus and bicarbonate production and a decrease in mucosal blood flow. Epigastric pain usually occurs within 15–30 min following a meal in patients with a gastric ulcer [3]. As a result of indiscriminate use of NSAIDs by people without prescription and not taking cognizance of what they eat, it accounts for over 90 % of all ulcers and approximately 25 % of NSAID users will develop peptic ulcer disease. Aspirin users are also twice as likely to develop peptic ulcers as the general population [4]. Despite the availability and use of most orthodox and conventional drugs, gastric ulcer still remains a global health challenge because they do not total undo the effect of NSAIDs. Also, not everybody likes taking the conventional drugs and not every patient can afford them. That is why gastric ulcer is still prevalent in most developing countries in which Nigeria is one of them [4]. Hence, the search for newer and efficacious approach to its management continues.

Yogurt is abundant in calcium, zinc, B vitamins, and probiotics; it is a good source of protein; and it may be supplemented with vitamin D and additional probiotics associated with positive health outcomes [5]. Probiotics are known to have beneficial roles in curing antibiotic associated diarrhea, constipation, traveler's diarrhea, food allergies and cancer [6]. According to Momoh et al. (2022), the consumption of unsweetened yogurts (containing probiotics) may improve glucose metabolism to a degree, but is more effective in treating gastrointestinal complications. The benefits of yogurt consumption to gastrointestinal function are most likely due to effects mediated through the gut microflora, bowel transit, and enhancement of gastrointestinal innate and adaptive immune responses [7]. We therefore seek to look at the antioxidative and anti-inflammatory effects of yoghurt in gastric ulceration. We also seek to determine the effective percentage concentration of the yoghurt on gastric ulceration.

## 2 Materials and methods

### 2.1 Drugs and reagents

Indomethacin and Omeprazole were purchased from Ningbo DHY Pharma. Co. Ltd. China. Pasteurized yoghurt was purchased from Uche care Pharmaceutical, Ondo State, Nigeria.

The instruments (dissecting set, pH meter, dissecting microscope, electrical weighing balance, plastic cages, e.t.c.) used in this study were obtained from the Department of Physiology, Faculty of Basic Medical Sciences, University of Medical Sciences, Ondo town, Nigeria and Federal University of Technology (FUTA), Akure, Ondo State, Nigeria.

### 2.2 Experimental animals

Thirty (30) rats weighing (100–150g) each were purchased from Mctemmy concepts laboratory animals for research and a disease-free animal farm in Osogbo, Osun state, Nigeria. The animals were kept in polypropylene plastic cages and maintained at normal and standard laboratory conditions of temperature (28 ± 2°C) and relative humidity (46 ± 6 %) with 12-h light-dark cycle and adequate ventilation for two weeks to allow for acclimatization to their new environment. The rats were weighed to ensure that none of them was outside their initial weight of 100–150g was lost. The entry point weight range was chosen to ensure that all the rats were matured enough for the laboratory experiment. The rats were fed with commercially available standard rat feed (Ladokun feeds Nig. Ltd) purchased from a commercial branch depot in Ondo city, Ondo state. They were given water ad libitum during the period of acclimatization. They were cared for and maintained in the animal facility in accordance with regulations, guidelines and policies governing the use of animals in research as described in public health service policy on human care and use of laboratory animals and approved by the University of Medical Sciences Animal Research Ethics Committee (UAREC) with approval number/ethical consent code UNIMED-AREC/Apv/2023/029.

### 2.3 Experimental design

The rats were divided into 5 groups of 6 animals each and pre-treated as stated below:

Group 1 (Control, n = 6): pre-treated with distilled water for 14 days

Group 2 (Indomethacin, n = 6): pretreated with distilled water for 14 days and thereafter administered with single dose of indomethacin (40 mg/kg) [8].

Group 3 (50 % yoghurt, n = 6): pretreated with 50 % diluted yogurt for 14 days and thereafter administered with single dose of indomethacin (40 mg/kg).

Group 4 (100 % yogurt, n = 6): pretreated with 100 % yogurt for 14 days and thereafter administered with single dose of indomethacin (40 mg/kg).

Group 5 (Omeprazole, n = 6): pretreated with omeprazole (20 mg/kg) [9] for 14 days and thereafter administered with single dose of indomethacin (40 mg/kg).

### 2.4 Ulcer induction

Gastric ulceration was induced in the animals according to the procedure described by Akpamu et al., 2013. Rats were administered with a single oral dose of indomethacin (40 mg/kg body weight). They were deprived of food but had free access to water 24 h prior to ulcer induction. Various degrees of ulceration have manifested 4 h after indomethacin administration.

### 2.5 Sample collection

After the period of the experimental procedures, the animals were euthanized by cervical dislocation after being fasted for 24 h with free access to water. Retro-orbital blood collection was done and kept in plain sample bottles to obtain serum for anti-inflammatory assays and the stomach was carefully dissected out. The stomach was thereafter divided into two, a part for histological evaluation and the other part for biochemical assays.

### 2.6 Ulcer scoring index (USI)

It is a complex process that involves evaluating the size, depth, tissue type, presence of necrosis, exudate, and surrounding skin condition of the ulcer, among other factors. The USI is based on several factors that are known to affect the severity and healing of gastric ulcers. These factors include the size of the ulcer, the depth of the ulcer, the presence of necrotic tissue, the type of tissue in the wound bed, the presence of exudate or drainage, and the surrounding skin condition. Each factor is assigned a score based on its severity, and the scores are added together to give an overall USI score. The USI scoring system is as follows:

• Size: Measure the length and width of the ulcer in centimeters. Multiply the length by the width to get the ulcer area. Assign a score based on the area of the ulcer:
  ✓ Less than 1 cm: 1 point
  ✓ 1–5 cm: 2 points
  ✓ 6–10 cm: 3 points
  ✓ More than 10 cm: 4 points
• Depth: Determine the depth of the ulcer. Assign a score based on the depth of the ulcer:

Once each category is evaluated and scored, the individual scores are added up to determine the total ulcer score. A higher total score indicates a more severe gastric ulcer [10].

### 2.7 Histological preparation and scoring

A part of the stomach tissue was kept in 10 % buffered formalin and thereafter embedded in paraffin. The sections (5 μm) were and stained with routine Hematoxylin and Eosin (H&E) protocol. The slides were observed under the light microscope and a semiquantitative scoring was done by modifying the scoring system of Turkyilmaz et al., 2019. The histological changes that occurred at the gastric mucosa were scored by observing the following; loss and architectural disruption in crypts, edema, vascular congestion and infiltration of polymorphonuclear cells and lymphocytes modified using a grading of normal, mild, moderate and severe and was given a score of 0, 1, 2 and 3 respectively.

Determination of Malondialdehyde (MDA), Superoxide dismutase (SOD), Catalase and Glutathione peroxidase in stomach tissues.

The stomach tissues prepared for biochemical assays were homogenized in Tris KCl buffer (pH 7.4) and thereafter centrifuged to obtain a clear homogenate. Lipid peroxidation assay was done by determining Malondialdehyde (MDA) activity according to the method of Varshney and Kale (1990). This method is based on the reaction between 2-thiobarbituric acid and MDA resulting in a pink solution which can be read at absorbance of 532 nm. Catalase (CAT) activity was performed according to the method of Sinha, 1971. The method is based on the reduction of dichromate in acetic acid to chromic acetate when heated in the presence of H_2_O_2_. The end product gives a deep green color which is read at 560 nm. Superoxide dismutase (SOD) activity was measured using the method described by Misra and Fridovich (1972). The method is based on the ability of SOD to inhibit the auto-oxidation of epinephrine at pH 10.2.

### 2.8 Total anti-oxidative capacity (TAC) in serum

Total antioxidant capacity was determined according to the method described by Rubio et al., 2016. The method employed the use of ferric reducing antioxidant power (FRAP) assay and the principle is based on the reduction of ferric-tripyridyltriazine complex (Fe^3+^-TPTZ) reagent to ferrous tripyridyltriazine (Fe^2+^-TPTZ) by the antioxidants present in the sample at low pH. Ferrous tripyridyltriazine (Fe^2+^-TPTZ) has a blue color and can be read at 593 nm.

### 2.9 Tumor necrosis factor-alpha (TNF-α), interleukin 10 (IL-10), Interleukin-1 beta (IL-1β) in serum

Tumor Necrosis Factor-alpha (TNF-α), Interleukin 10 (IL-10) and Interleukin 1 Beta (IL-1β) were quantified using enzyme-linked immunosorbent assay (ELISA) kits purchased from Elabscience, Houston, Texas, United state and the manufacturer's instruction followed appropriately.

### 2.10 Statistical analysis

Data was analyzed using statistical software; Graphpad prism (version 9). Statistical significance was assessed using oneway analysis of variance (ANOVA) with Turkey (Post hoc test). P values of <0.05 were considered statistically significant. Results (all mean values) were expressed as group Mean ± SEM (Standard Error of Mean).

## 3 Result

### 3.1 Effect of on gross appearance and ulcer score

**Fig. 1:**
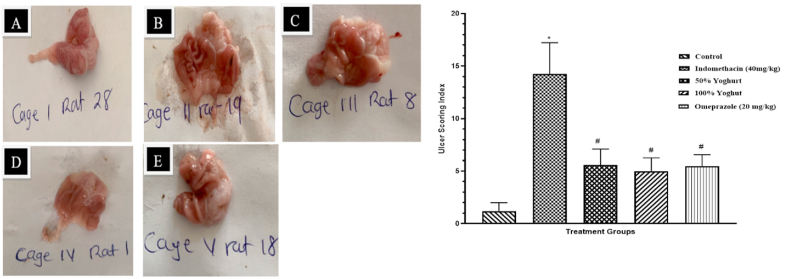
Effect of Yoghurt on gross morphology and ulcer score A: Control; B: Indomethacin; C: 50 % Yoghurt; D: 100 % Yoghurt; E: Omeprazole (40 mg/kg). Values are expressed as Mean ± SEM (n = 6). ∗significant when compared to Control. ^#^significant when compared to Indomethacin (40 mg/kg).

The control group showed normal mucosa with no visible lesion. The administration of Indomethacin resulted in severe ulcer in indomethacin group compared resulting in a very high ulcer scoring index the control group. The ulcer scoring index was significantly decreased in both Yoghurt groups compared to indomethacin group as the both showed mild ulceration (see Fig. 1).

### 3.2 Effect of yoghurt on histological observations and score

**Fig. 2:**
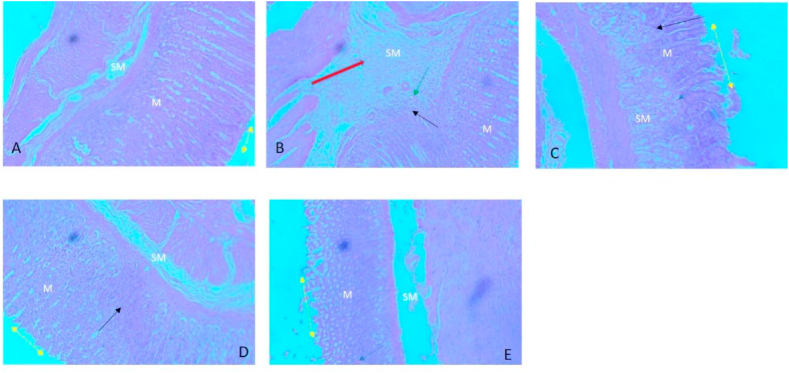
Photomicrograph of stomach for respective groups X100. **A** (Control) All tunics appear intact and normal. Submucosa (SM) and Mucosa (M). **B** (Indomethacin 40 mg/kg) Severe architectural disruption of crypt. Severe inflammation and infiltration of inflammatory aggregates up to the submucosa (Black arrow). Edema in the submucosa (Red arrow) and vascular congestion (green arrow). **C** (50 % Yoghurt) Moderate erosion of epithelium of mucosa (yellow spanning arrow). Other tunics appear normal. Mild infiltration of inflammatory aggregates at the mucosa layer (black arrow). **D** (100 % Yoghurt) Mild erosion of epithelium of mucosa (yellow spanning arrow). Other tunics appear normal. Mild infiltration of inflammatory aggregates at the mucosa layer (black arrow). **E** (Omeprazole 20 mg/kg) Mild erosion of epithelium of mucosa (yellow spanning arrow) and vascular congestion (green arrow). Other tunics appear normal. Mild infiltration of inflammatory aggregates at the mucosa layer (black arrow).

**Fig. 3:**
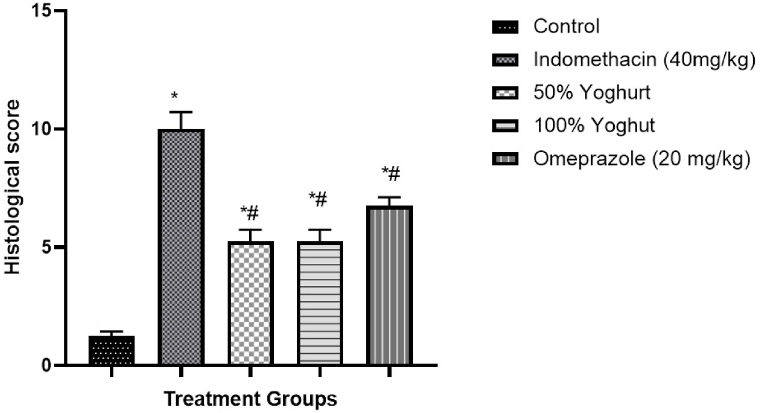
Effect of Yoghurt on Histological score. Values are expressed as Mean ± SEM (n = 6). ∗significant when compared to Control. ^#^significant when compared to Indomethacin (40 mg/kg).

Histological sections revealed normal architecture of the crypts in the control group from the mucosa to the serosa layers. However, severe crypt disruption, edema and infiltration of inflammatory aggregates were observed in indomethacin (40 mg/kg) group following indomethacin administration. These changes were mild in yoghurt groups and moderate in omeprazole group as shown in Fig. 2. Fig. 3 furthermore showed an increase in histology score in indomethacin (40 mg/kg) group compared to control group. This score was significantly reduced in both yoghurt groups and omeprazole group.

### 3.3 Effect of yoghurt on biochemical parameters and inflammatory markers

**Fig. 4:**
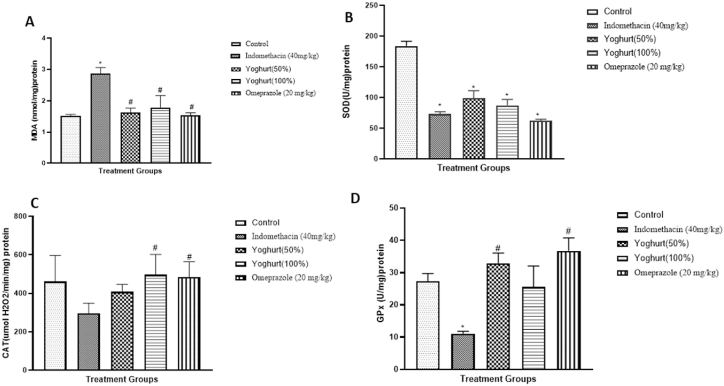
Effect of Yoghurt on biochemical assays in indomethacin induced gastric ulceration. A: Malondialdehyde (MDA); B: Superoxide dismutase (SOD); C: Catalase (CAT); D: Glutathione Peroxidase. Values are expressed as Mean ± SEM (n = 6). ∗significant when compared to Control. ^#^significant when compared to Indomethacin (40 mg/kg).

Effect of yoghurt on biochemical parameters is depicted in Fig. 4. Malondialdehyde (MDA) was significantly increased in indomethacin grouped compared to control group. Treatment with yoghurt and omeprazole significantly reduce MDA compared to indomethacin group. Superoxide dismutase (SOD), Catalase (CAT) and Glutathione peroxidase (GPx) where all significantly decreased after indomethacin administration in the indomethacin group compared to control. Yoghurt treatment as well as omeprazole induction significantly reverse CAT and GPx but not SOD.

**Fig. 5:**
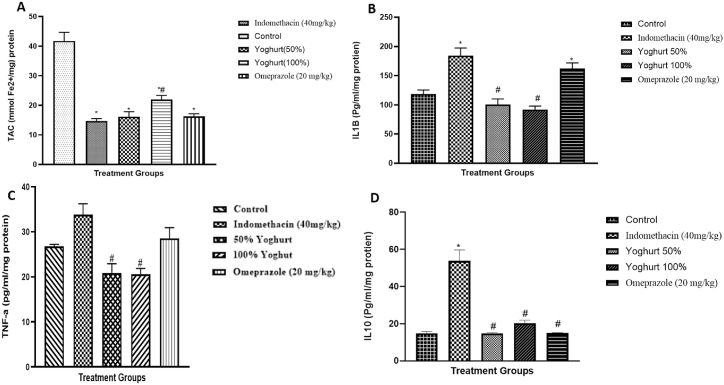
Effect of Yoghurt on inflammatory markers in indomethacin induced gastric ulceration. A: Total antioxidant capacity; B: Interleukin 1β; C: Tumor necrosis factor-α; D: Interleukin 10). Values are expressed as Mean ± SEM (n = 6). ∗significant when compared to Control. ^#^significant when compared to Indomethacin (40 mg/kg).

Inflammatory markers are shown in Fig. 5. Interleukin 1- beta, interleukin 10 and tumor necrosis factor alpha were all significantly increased in indomethacin (40 mg/kg) group compared to control group. Yoghurt groups significantly decreased these parameters compared to indomethacin (40 mg/kg) group. Furthermore, total antioxidant capacity was significantly higher in 100 % yoghurt group and control group compared to indomethacin (40 mg/kg) group. Omeprazole (20 mg/kg) and 50 % yoghurt groups showed no significant difference compared to indomethacin (40 mg/kg) group.

## 4 Discussion

Our study has shown that consumption of pasteurize yoghurt for 2 weeks was able to boost the immune system by decreasing inflammatory markers and decrease oxidative stress via enhancement of antioxidant enzymes in the stomach against indomethacin induced gastric ulceration. Peptic ulcer is the most prevalent gastrointestinal disease [10] and has long been reported to occur when the gastro-aggressive factors overwhelm the gastroprotective factors [11].

Administration of indomethacin successfully induce gastric mucosa lesion evident in gross observation of the stomach and high ulcer index of the indomethacin group. Pre-treatment with both concentrations of yogurt (50 and 100 %) resulted in a remarkable decrease in gastric mucosa damage and ulcer index that is comparable to the standard drug administered (omeprazole). Previous report has shown similar effect of yogurt in acetic acid-induced gastric ulcer where yogurt containing *lactobacillus gasseri* promote gastric ulcer healing [12]. Also according to Momoh et al. (2022), it was observed that administration of the yogurt caused cell infiltrations of the stomach wall, distinct ductile without aberrant superior or inferior diverticular of the stomach interlobular lining and well-formed stomach blood vessels.

Histological observations from this study also showed deep ulcer and severe inflammation as a result of increase infiltration of inflammatory cells into the stomach. Yogurt exert cytoprotective effect on indomethacin induced gastric ulcer by decreasing gastric mucosa erosion and reducing infiltration of inflammatory aggregates. This action was more effective in the 100 % yogurt compared to the other treated groups. Yogurt has been reported to have cytoprotective effects on the gastric gland against HCl-induced acute gastric lesions and this action was exerted via their ability to promote prostaglandin production [13]. Prostaglandins are important in the stomach to enhance blood flow and for the production of mucus and bicarbonates. Indomethacin is a potent cyclooxygenase blocker thus; the administration of indomethacin will inhibit COX which is the enzyme that catalyzes the production of prostaglandin. This will lead to decrease in prostaglandin which in turn will lead to reduced gastric mucosa blood flow, mucous and bicarbonate production as well as increase acid production thereby promoting ulcer formation [1,13]. Consequently, the gastroprotective action of yogurt observe in this study can be linked to its ability to promote prostaglandin production.

Oxidative stress occurs as a result of imbalance between oxidants (free radicals) and antioxidant enzymes in favor of the former, which then causes damage to tissues. It is usually associated with indomethacin induced gastric ulcer model [14,15]. One major end product of lipid peroxidation is malondialdehyde (MDA) which has been widely used as a marker of oxidative stress in several disease conditions such as cardiovascular, gastrointestinal and neurodenerative disease [2,16,17]**.** In this study, the level of MDA was significantly higher in the indomethacin group compared to control indicating that the administration of indomethacin resulted in lipid peroxidation and this is consistent with other reports [14]. There was a corresponding decrease in antioxidant enzymes; superoxide dismutase-SOD, catalase- CAT and glutathione peroxidase- GPx. Yoghurt pretreatment resulted in a significant restoration of MDA level to control value and increase in the antioxidant enzymes which suggest that yoghurt has antioxidant properties. It has been reported by several studies that probiotics have direct antioxidative activities and can also modify the immune system [18]. The effect of yoghurt on MDA could be as a result of its probiotic properties. The lactic acid bacteria present in yogurt has direct ability to scavenge free radicals and also suppress their generation [18]. Thus, preserving the body's antioxidants defense mechanism as noted in the significant increase in antioxidants enzymes compared to indomethacin group. Superoxide dismutase (SOD) which serve as first line defense against reactive oxygen species by converting the highly reavtive O2- to a less reavtive H2O2 which is further mopped up by the actions of catalase (CAT) and glutathione peroxidase- (GPx). Furthermore, the total antioxidant capacity which measures the activities of the non-enzymatic component of the antioxidant defense mechanism was also enhanced in the yogurt treated groups.

The infiltration of inflammatory aggregates such as macrophages as well as the generation of reactive oxygen species have been reported to activate pro inflammatory cytokines especially tumor necrosis factor alpha (TNF-α) and Interleukin 1 beta (IL1β) [19]. These inflammatory cytokines are closely associated with gastric ulcer [19,20] indicating the severity of the disease condition. TNF-α and IL1β production are very high at the inflammatory phase of gastric ulcer which usually decreases as healing progress [21]. Interleukin 10 is usually considered as an anti-inflammatory cytokine. It was initially noted to be a product of the T helper 2 cells which inhibits the activation of T helper 1 cell. However, it is now known to be produced by several immune cells [22]. Interleukin 10 regulates immune responses and maintains immune homeostasis and its reduction indicates a decrease in inflammatory process [23].

## 5 Conclusion

In conclusion, this study has observed the effect of pasteurized yogurt on gastric ulcer by assessing oxidative stress parameters, pro- and anti-inflammatory cytokines as well as histological appearance. The study demonstrated that pasteurized yogurt exerts anti-oxidative and anti-inflammatory effect in indomethacin-induced ulcerated male Wistar rats, as indicated by the decrease in IL-10, IL-1B, TNF-a, and increase in TAC level and a significant difference in the anti-oxidative parameters. These findings support the potential of pasteurized yogurt as a dietary intervention for oxidative and inflammation-associated conditions.

## CRediT authorship contribution statement

**O.V. Ajayi:** Writing – original draft, Resources, Project administration, Methodology, Funding acquisition, Conceptualization. **B.O. Omolaso:** Writing – review & editing, Methodology. **T.P. Omayone:** Writing – review & editing, Formal analysis. **A.G. Adeniran:** Writing – review & editing. **A.O. Adu:** Writing – original draft, Data curation. **O.E. Olorunfemi:** Writing – original draft, Data curation.

## Compliance with ethical standards

The experimental procedures were approved by the UNIMED Animal Care and Use Research Ethics Committee and performed in accordance with the care and use of Laboratory Animals of the NIH Guidelines by careful handling.

## Limitations of the study

● The disease model used in this study, though suitable for induction of gastric ulcer can only be used to detect the cytoprotective action and cannot be used for ulcer healing.

## Ethical statement

Ethical approval for this study was obtained from the University of Medical Sciences Animal Research Ethics Committee (UAREC) with approval number/ethical consent code UNIMED-AREC/Apv/2023/029 and the study complies with all regulations. Authors declare that they have no conflict of interest.

### Consent to participate

All authors gave their consent to participate in this study.

### Consent to publish

All authors consented to the publication of this article.

## Funding

The study was done with ADU and OLORUNFEMI financial contribution and did not receive any sponsorship from any agency.

## Declaration of competing interest

The authors declare that they have no known competing financial interests or personal relationships that could have appeared to influence the work reported in this paper.
